# Atypical posterior scleritis mimicking an amelanotic choroidal melanoma. A case report

**DOI:** 10.22336/rjo.2021.57

**Published:** 2021

**Authors:** Maria Klecheva Maksimova, Elisabet Martín García, Irune Ortega Renedo, José Javier Chavarri García, Beatriz Jiménez Del Río, Leticia Rodríguez Vicente, José Luis Del Río Mayor

**Affiliations:** * Department of Ophthalmology, San Pedro Hospital, Logroño, Spain

**Keywords:** posterior scleritis, choroidal melanoma, differential diagnosis, autoimmune, multidisciplinary

## Abstract

**Purpose:** To describe a clinical case of atypical posterior scleritis mimicking an amelanotic choroidal melanoma.

**Method:** Observational case report of a 54-year-old woman who presented to the emergency department with photophobia and blurred vision in her left eye for three days. The development of a raised hypopigmented lesion superior to the papilla with choroidal folds and without vitritis simulated an amelanotic choroidal melanoma. Differential diagnosis took into consideration other compatible entities, including choroidal masses or orbital pseudotumor.

**Results:** The patient was subject to full clinical examination, laboratory test, optical coherence tomography, orbital echography, and magnetic resonance imaging. Treatment with oral prednisone showed a significant improvement in all clinical and anatomical parameters.

**Discussions:** Posterior scleritis is characterized by great clinical variability and sometimes can simulate an amelanotic choroidal melanoma. Performing an appropriate differential diagnosis of a large amelanotic lesion is the most important point during a routine ocular examination due to the implications for the patient.

**Conclusions:** Posterior scleritis is a rare and incompletely understood inflammatory disease that affects the posterior part of the sclera. It can be associated with a range of conditions and very often is underdiagnosed. In about one third of the cases, it is related to some systemic disease, especially to autoimmune entity, so it may require a multidisciplinary approach. This case highlighted the importance of a solid differential diagnosis and an early treatment in order to help prevent the appearance of complications that can limit not only the visual outcome of the patient but even his survival in the most extreme cases.

**Abbreviations:** LE = left eye; RE = right eye; BCVA = best corrected visual acuity; BO = both eyes; IOP = intraocular pressure; OCT = optical coherence tomography; MRI = Magnetic Resonance Imaging

## Introduction

Posterior scleritis is an inflammatory disease that affects the sclera located behind the ora serrata. It is a rare entity with a prevalence of 6 cases per 100,000 persons that mainly affects women between 30 and 60 years old. It is usually an idiopathic condition, although between 19.5 and 37% of cases are associated with systemic diseases such as rheumatoid arthritis, granulomatosis with polyangiitis or systemic lupus erythematosus [**1**,**2**].

Clinically, patients report the sudden onset of periocular pain, binocular diplopia, decreased visual acuity, proptosis and eyelid swelling. Other clinical findings may include an exudative retinal detachment, choroidal folds, subretinal fluid, macular edema, papilledema and even a tumor-like nodular mass [**3**,**4**]. 

We described an uncommon case of a patient with posterior scleritis, which simulated an amelanotic choroidal melanoma.

## Case report

A 54-year-old woman with no significant medical history presented to the emergency department with photophobia and blurred vision in her left eye (LE) for three days. The best-corrected visual acuity (BCVA) was 20/ 20 in both eyes (BO) and the intraocular pressure (IOP) was normal. A slit lamp examination revealed the presence of mild temporal conjunctival hyperemia associated with chemosis in the LE. The funduscopic examination was unremarkable. Topical treatment with Diclofenac 1 mg/ ml every 8 hours was started in her LE.

**Fig. 1:**
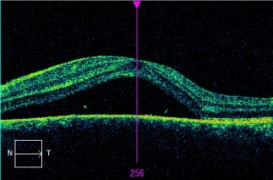
Macular OCT. Exudative retinal detachment

Four days after she came to the emergency department again with a significant decrease in her VA and the onset of proptosis in the LE. The BCVA was 20/ 20 in her right eye (RE) and 10/ 20 in her LE. Chemosis persisted and fundus examination revealed the presence of a raised hypopigmented lesion superior to the papilla with associated choroidal folds without vitritis. The macular optical coherence tomography (OCT) showed a wide neurosensorial detachment (**Fig. 1**). 

**Fig. 2:**
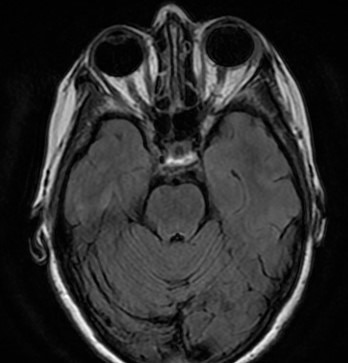
Orbital magnetic resonance image. Posterior scleral thickening and proptosis of the LE

Suspecting an amelanotic choroidal melanoma, a B-mode ultrasound was performed, showing hyperreflectivity and a thickening of the posterior sclera. In addition, a brain and orbit Magnetic Resonance Imaging (MRI) revealed a slight posterior choroidoscleral thickening in the LE with Gadolinium uptake in T2 and 3 mm ocular protrusion (**Fig. 2**). Autoimmune, biochemical, and serological tests were normal.

Treatment with oral prednisone 60 mg / day was initiated. A week after, the patient showed a significant improvement in all clinical and anatomical parameters. 

**Fig. 3:**
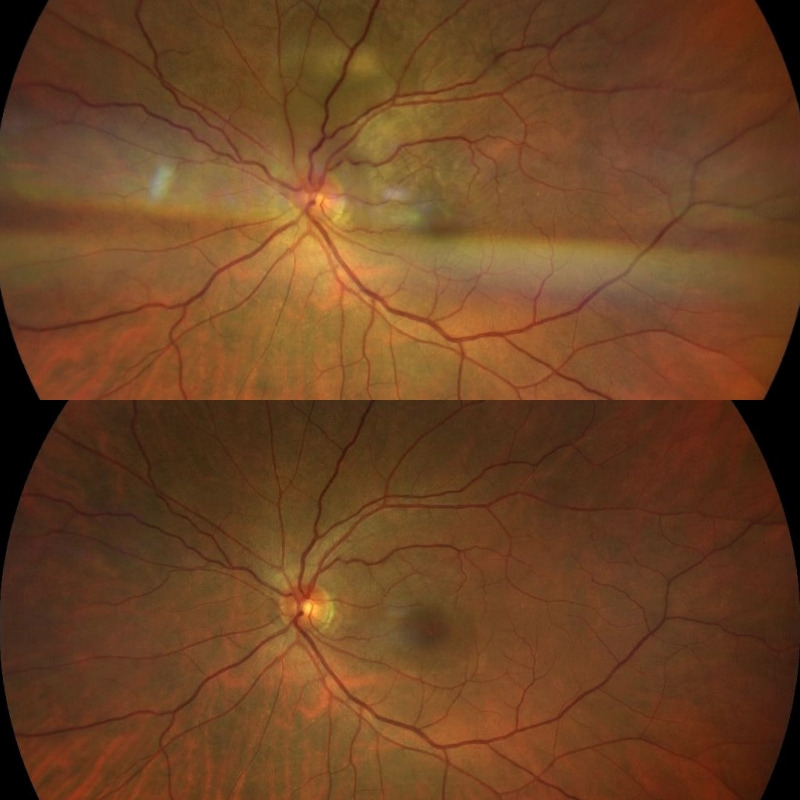
Retinography of the LE showing a large choroidal non-pigmented lesion superior to the optic disc and the recovery after treatment with systemic corticosteroids

## Results

A week after, the patient showed a significant improvement in all clinical and anatomical parameters. After 20 days, progressive decrease in oral corticosteroids was started due to the experienced improvement in her clinical and functional state, achieving stability in successive revisions (**Fig. 3**). The progressive reduction of oral corticosteroids was then started until achieving stability in successive revisions.

## Discussion

Posterior scleritis is characterized by great clinical variability in its presentation. In our case, the condition was atypically manifested as a painless subretinal hypopigmented mass and underlying scleral inflammation [**5**]. 

It is important to highlight that in up to 64% of the cases, posterior scleritis presents periocular pain as the main symptom, which increases with the eye movements and is radiated to the cranial region as a result of compression and stimulation of the trigeminal nerve. This fact can be mistaken with other neurological diseases contributing to its under and misdiagnosis. However, painless presentations have been shown in previous studies like in the case of our patient [**6**].

Decreased visual acuity can be caused by macular involvement, optic neuritis and even by hyperopia due to the flattening induced by the inflamed sclera, which implies a reduction in axial length manifested in the form of choroidal folds at the funduscopic examination.

Several studies highlighted the importance of evaluation using B-mode ultrasound, considering it the most relevant imaging method for diagnosing posterior scleritis [**7**]. Likewise, magnetic resonance imaging of the orbit is very useful in differentiating a posterior scleritis from a choroidal tumor mass or an orbital pseudotumor. In our case, the combination of both diagnostic tests allowed us to establish the diagnosis of posterior scleritis [**8**]. 

The visual outcome depends on the duration of the condition, its intensity and the associated complications, so prompt recognition before starting the treatment is essential. A high index of suspicion can avoid the appearance of complications that may threaten not only the patient’s vision but also his life, as ocular manifestations can be the initial presentation of life-threatening illness.

Therapeutic options depend on the inflammation degree. Systemic corticosteroids (especially the oral form) are considered the first-line treatment. A therapeutic approach for the most severe cases involves intravenous methylprednisolone 1 g/ per day for 3 days followed by oral corticosteroids. Another option is the subconjunctival administration of corticosteroids, which are highly effective and avoid systemic side effects; however, they are contraindicated in necrotizing scleritis due to the risk of scleral thinning and subsequent ocular perforation. In the most severe and resistant cases, immunosuppressive drugs such as azathioprine, cyclosporine, and cyclophosphamide can be a perfect therapeutic alternative [**9**,**10**].

## Conclusion

In conclusion, posterior scleritis can pose a diagnostic challenge for ophthalmologists and may require a multidisciplinary approach. This case shows how the importance of differential diagnosis and the prompt disease recognition become a therapeutic significance to avoid ophthalmological and systemic complications and to maintain the anatomical and functional integrity.

**Conflict of Interest statement**

The authors state no conflict of interest.

**Informed Consent and Human and Animal Rights statement**

Written informed consent was obtained from patient to publish findings and images presented in this manuscript.

**Authorization for the use of human subjects**

Ethical approval: The research related to human use complies with all the relevant national regulations, institutional policies, is in accordance with the tenets of the Helsinki Declaration, and has been approved by the review board of San Pedro Hospital, Logroño, Spain.

**Acknowledgements**

None.

**Sources of Funding**

The author(s) received no financial support for the research, authorship, and/ or publication of this article.

**Disclosures**

The author(s) declare no potential conflicts of interest with respect to the research, authorship, and/ or publication of this article.
